# Engineered Tumor Cell Apoptosis Monitoring Method Based on Dynamic Laser Tweezers

**DOI:** 10.1155/2014/279408

**Published:** 2014-04-01

**Authors:** Yuquan Zhang, Xiaojing Wu, Changjun Min, Siwei Zhu, H. Paul Urbach, Xiaocong Yuan

**Affiliations:** ^1^Key Laboratory of Optoelectronic Information Science & Technology, Institute of Modern Optics, Nankai University, Ministry of Education of China, Tianjin 300071, China; ^2^Institute of Oncology, Tianjin Union Medicine Centre, Tianjin 300121, China; ^3^Optics Research Group, Delft University of Technology, Lorentzweg 1, 2628 CJ Delft, The Netherlands; ^4^Institute of Micro & Nano Optics, Shenzhen University, Shenzhen 518060, China

## Abstract

Monitoring the cells' apoptosis progression could provide a valuable insight into the temporal events that initiate cell death as well as the potential for rescue of apoptotic cells. In this paper, we engineered a novel and robust method for monitoring apoptosis of tumor cells based on dynamic laser tweezers, using A549 and HeLa cell line as typical samples. The entire experiment can be completed in a few hours with small amount of fluid sample, presenting great advantages of celerity, microscaled measurement, and label-free explorations without perturbing experimental conditions in combination with other probes. Validity and stability of this method are verified experimentally in terms of physical parameters of the system. The proposed technique has great potential in improving cancer treatment by monitoring the objective efficacy of tumor cell killing.

## 1. Introduction

Apoptosis [1, 2], as the process of programmed cell death, occurs in multicellular organisms. Ever since it was discovered 40 years ago, apoptosis remains one of the most investigated processes in biological research [1]. As a highly selective process, it is important in both physiological and pathological conditions, while in cancer there is a loss of balance between cell division and cell death, where cells that should have died may not receive the signal to do so [3, 4]. Consequently, apoptosis monitoring in cancer conditions is vitally important for cancer treatments, because it may assess the effectiveness of the potential therapeutic interventions [5, 6].

For apoptosis monitoring, conventional monitoring technologies generally take anatomical imaging approaches to detect the late-stage abnormalities and thus are always labeled essential and time consuming [7–9]. As an important novel alternative to conventional techniques, optical technology offers advantages of label-free detection and monitoring the early changes in anticancer therapy [10–12]. Optical tweezers [13], typically in an exponential relationship with refractive indices of cells, have been proved a powerful tool in the investigation of individual cell properties, such as deformation, stretching, folding, and rotation [14–20].

In this study, we proposed a reliable and reproducible method for monitoring apoptosis based on dynamic laser tweezers and established two cell lines, A549 and HeLa, to experimentally demonstrate this method. Compared to conventional methods with disadvantages of expensive, time-consuming, complicated reaction process, and requirement of viable tumor cells, the proposed method shows advantages of label-free, fast process (within hours) and microscaled measurement (dozens of microliter in microflow channel) for monitoring apoptosis quantitatively in terms of the values of optical trapping efficiency at different drug concentration levels. We also verified its validity and stability by comparing with the flow cytometer approach which is previously routinely used in biomedical laboratories. The results indicate great potential of our proposed method in the studies of apoptosis monitoring with high sensitivity and efficiency.

## 2. Experiment

### 2.1. Sample Preparation

We established samples of apoptotic cell lines caused by the concentration of cisplatin, which is a well-known chemotherapy drug used to treat cancers including sarcoma, small cell lung cancer, germ cell tumors, lymphoma, and ovarian cancer [21–23]. Cisplatin functions as a noncell cycle specific, bifunctional, alkylating agent, as it contains no alkyl groups and does not instigate alkylating reactions. The way it operates is forming a platinum complex inside of a cell which binds to DNA and cross-links DNA. One of the methods by which it causes apoptosis through cross-linking is damaging the DNA, so that the repair mechanisms for DNA are activated, and then the cells are found not to be salvageable; thus, the death of those cells is triggered instead. It has revolutionized the treatment of carcinoma, making a previously deadly disease potentially curable. In this work, the HeLa and A549 cells (purchased from the Type Culture Collection of the Chinese Academy of Sciences, China), which are frequently used in scientific research, were chosen in experiments of apoptosis monitoring due to their superior sensitivity to cisplatin. A549 cells were cultured in RPMI 1640 medium (and HeLa in DMEM) supplemented with 10% fetal bovine serum (FBS), penicillin-G (100 U/mL), and streptomycin (100 mg/mL). Then the cells were exposed to 3 different concentrations of cisplatin (3 *μ*g/mL, 10 *μ*g/mL, and 30 *μ*g/mL), and after incubation for 24 hours, the apoptosis level was detected by flow cytometry (FCM) for comparison with the apoptosis monitoring result of laser tweezers.

### 2.2. Experiment Setup

**Figure 1:**
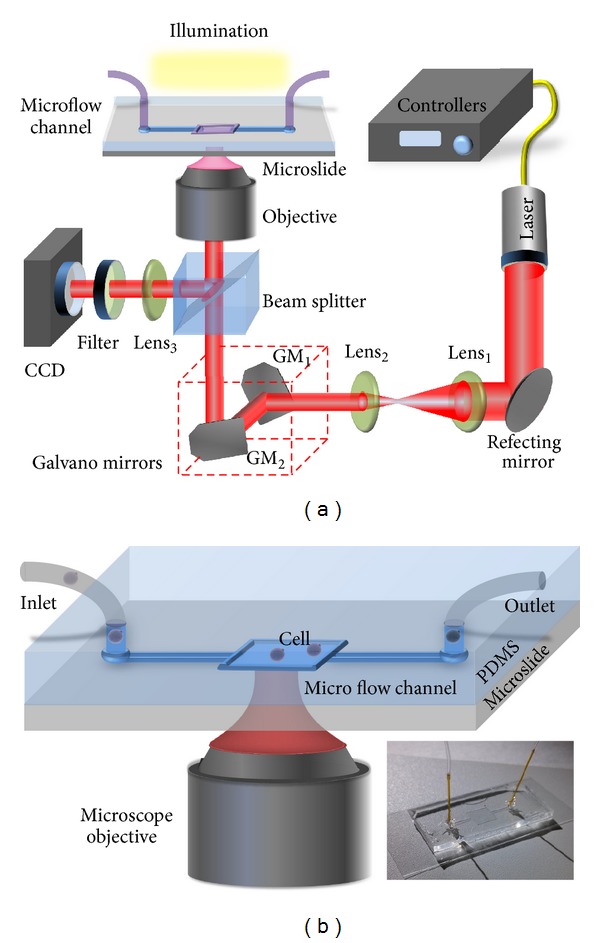
Schematic of the laser tweezers system that generates dynamic steering traps (a). Structural distribution of microfluidic chip; inset is the device photograph (b). GM: galvanometer mirror.

The laser tweezers experimental setup is shown in Figure 1(a). The light source is a 5 W power, 1070 nm wavelength Yb fiber laser (IPG Laser Gmbh), whose output intensity can be tuned with a step of 50 mW. The beam passes through a conjugated lens pair (lens_1_ and lens_2_) to pinch the beam size to fit the entrance of the objective (Nikon, 100× oil immersion, N.A. = 1.25). Galvanometers (GM_1_ and GM_2,_ Cambridge Technology Inc.) are used as beam positioning and steering elements to make the laser beam moving along a dynamic circular trajectory before entering the inverted microscopic objective; thus, the trapped cell will follow the circular trajectory with a demagnified radius around the optical axis continuously. A white light source illuminates the samples from the top; the sample movement is finally imaged on a CCD camera.

Trapping manipulations on the apoptotic sample cells are performed in a plain microfluidic chip which is illustrated in Figure 1(b), employing several microliter samples for each measurement. The chip has a square sample chamber in the center and input and output rib channel ports on opposite sides. The side length of sample chamber is 5 mm, and each rib channel has dimensions of 8 mm in length, 1 mm in width, and 100 *μ*m in depth. Inlet and outlet holes were opened by a punch and cell solution is pumped into the channel through microcatheter with 0.435 mm external diameter and 0.320 mm inner diameter. Fluid sample is pumped into the microflow channel through inlet catheter by means of a syringe and a syringe pump. Cells suspend in the quadrate cuvette when pumping stops, and the one near the focus will be trapped by the focused laser beam. The trapped cell then moves along the trajectory of the laser regulated by the galvanometers.

### 2.3. Dynamic Operation Principle

It is well know that moving cell in aquatic environment receives a viscous resistance force, which is proportional to the speed. The force will stop the cell's circular motion when the speed increases to the critical escape velocity, and then the cell will escape from the optical trap. In our system, microscope's working distance is 210 *μ*m and thickness of the microslide is 170 *μ*m; thus, there exists at least a 20 *μ*m gap between the trapped cell and substrate and a 60 *μ*m interval to the channel roof. As a consequence, the cell in the laser tweezers could be considered lying in a free aquatic environment approximately, which supports the approximate hypotheses of considering the trapped cell as spherical. Then the optical trapping force for a spherical particle can be calculated by [24]

(1) $$\begin{array}{c} F_{\text{trap}}=\frac{nQP}{c}, \end{array}$$

where *P* is the trapping laser power, *n* is refractive index of the surrounding medium, *c* is the speed of light in vacuum, and *Q* is the optical trapping efficiency as a dimensionless parameter only depending on the trapping force onto the cell. Spherical particles moving slowly in a viscous fluid encounter a resistance force, within the Stokes flow limit, to be derived as [25]

(2) $$\begin{array}{c} F_{\text{drag}}=6\pi \eta av, \end{array}$$

where **η** is the viscosity of the fluid, *a* is the radius of the particle, and *v* is the rotating velocity.

According to (2), the drag force is proportional to the rotating velocity; thus, when the cell reaches a critical escaping velocity, the drag force is equal to the optical trapping force; that is,

(3) $$\begin{array}{c} F_{\text{trap}}=6\pi a\eta v_{\text{critical}}=F_{\text{escape}}=\frac{nQP}{c}. \end{array}$$

Consequently, we get

(4) $$\begin{array}{c} Q=\frac{6\pi \eta acv}{nP} \end{array}$$

which means that in the case of critical escaping velocity the trapping efficiency *Q* is dependent on trapped cell's radius *a*, refractive index of the surrounding medium *n*, trapping power *P*, viscosity coefficient **η**, and critical escaping velocity *v*.

In this work, the parameters *n* and **η** are constant; the cell's radius *a* has negligible effect on trapping efficiency *Q* as experimentally demonstrated in previous works [26]. Thus the trapping efficiency *Q* is determined by the ratio *v*/*P*. Since the critical escaping velocity has a linear relationship with the incident power value [27], the ratio *v*/*P* should be a constant at different incident power *P*. For a fixed incident power *P*, we increased the rotating velocity by software (EzCad2, JCZ Technology Co., Ltd.) to record the corresponding escaping velocity and repeated this process several times at different *P* to provide statistical data for the calculation of trapping efficiency *Q*. The trapping power lies in 10–30 mW in experiments, and the velocity was slow enough to ignore the vortex induced by the cell revolving. Consequently, at a fixed power *P*, the only factor that leads to the diversity of escape velocity was the different refractive indices of apoptotic cells induced by cisplatin. In other words, the cell apoptosis induced by cisplatin can be monitored by the escape velocity as well as the trapping efficiency *Q* in such laser tweezers system.

In experiment, the ambient temperature was scrupulously kept at 25°C to confirm the accuracy of experimental results (ignoring the weak heating effect by the laser beam). Cell samples were diluted for fifty times by normal saline. After the dilution, the dynamic viscosity value of surrounding medium was approximated as 1.0 × 10^−3^ N·s/m^2^ and the refractive index of surrounding medium as 1.33 (normal saline) for simplicity, and mean value of trapping efficiency for dozens of individual cells under each concentration was used to eliminate individual differences and get statistical significance. Diameters of trapped cells in our experiment distribute randomly in the range of 12–20 *μ*m. The trapping efficiency is a macroscopic presentation by considering the whole cell as one research subject, while cell apoptosis is a complex biochemical change process (i.e., damaging the DNA) and will lead to refractive index changes to influence the trapping efficiency.

## 3. Results and Discussions

**Figure 2:**
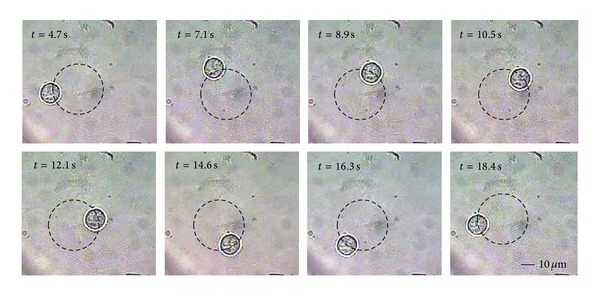
Successive frames of a video recording that show a single A549 cell rotating along a clockwise circumference trace.

Figure 2 shows the CCD captured video screenshots of the trapped cell with a diameter of 15 ± 0.5 *μ*m rotating along a circumference trace in isotonic buffer. The EzCad2 software is used to design the motion path and keep on updating the velocity till the cell reached the critical escaping velocity.

**Figure 3:**
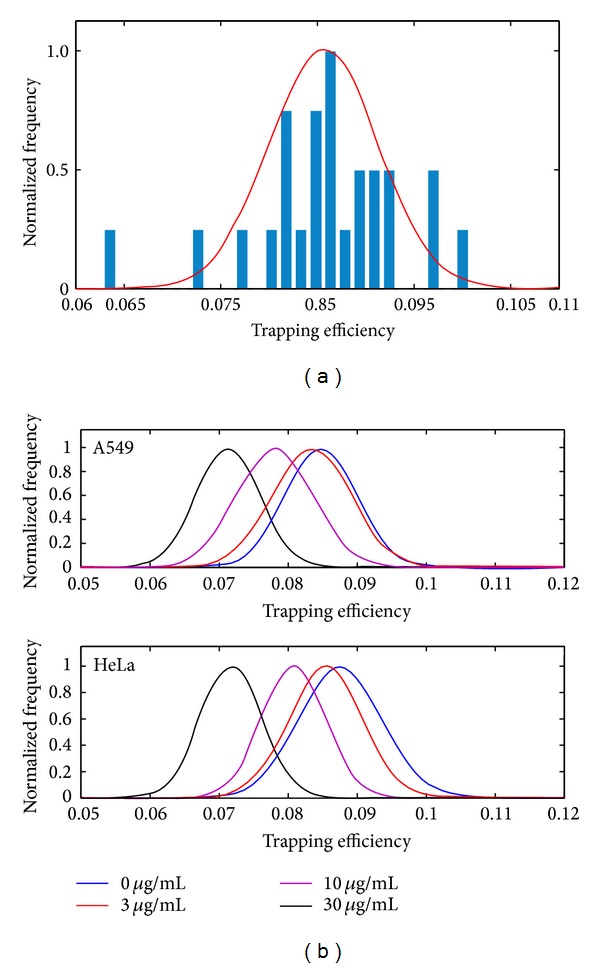
Fitted Gaussian curve and measured trapping efficiency of normal A549 cell (a) and fitted curves of A549 and HeLa cells with incremental cisplatin concentrations (0, 3, 10, 30 *μ*g/mL) (b).

**Figure 4:**
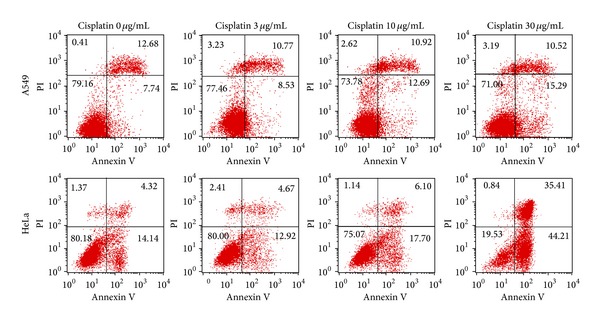
Flow cytometer results of A549 and HeLa cells. The four columns represent the cells' biological activity under cisplatin concentration annotated above.

Dozens of independent cells were measured for each concentration of cisplatin, and calculated results would exert deviation due to individual difference and apoptosis degree.  Figure 3 shows the trapping efficiency of the two cell lines with incremental cisplatin concentrations (0, 3, 10, and 30 *μ*g/mL) after 24-hour cultivation.  Figure 3(a) is the fitted Gaussian curve (red line) of normal A549 cells, nearest to the shape of the histogram (measured *Q*-parameters), and Figure 3(b) demonstrated the shift of fitted curves under the corresponding four concentrations. In Figure 3(b), the horizontal axis demonstrates the trapping efficiency calculated from our experimental data, and the vertical coordinate is the normalized frequency proportion that corresponding trapping efficiency occupies in whole measurements. Simultaneously, flow cytometer results are exhibited in Figure 4 as comparison, where cells were treated with Annexin V-FITC and propidium iodide (PI), to verify the veracity of our method. It shows that the apoptosis index increased with the increasing of cisplatin concentration.

Figure 3(b) shows obviously that the fitted curves move in the direction of the negative axis with increased cisplatin concentration, accompanied with the proportion of apoptosis escalating for both cell lines (as shown in Figure 4). For both cell lines, the trapping efficiency decreases smoothly with increasing cisplatin concentration at low level (from 0 to 3 *μ*g/mL). In order to verify its accuracy, statistical testing of hypothesis (*t*-test) was made on these results. However, result of the two groups shows that the difference at such low level is not really distinguishable, which shows excellent agreement with the FCM experimental results, because the apoptosis index did not increase obviously in such low level. While for higher levels (from 3 to 30 *μ*g/mL), the trapping efficiency of A549 still decreases smoothly, but that for HeLa cell decreases acutely on account of the higher sensitivity to cisplatin.* t*-test results indicate that it has significant difference under such concentrations by standard method at a significant level of 5%, which is confirmed by the FCM experimental results in Figure 4.

This laser tweezers based method operates the cell via a noncontact mode and no label is needful; thus, the trapped cell could remain metabolically active till the end of experiment, and each measurement takes only a few minutes. The experimental results verify that the capability of proposed method in detecting antitumor drug caused slight variation of the cells. Taking advantages of this system, cell apoptosis in cancer therapeutic process can be recognized rapidly and effectively, while it is crucial in clinical scientific researches. Therefore, this novel approach in assessing apoptosis could help clinicians to detect cancer therapeutic status to cisplatin (not just limited to such drug) and determine a more appropriate line of therapy for patients with cancer. Furthermore, such laser tweezers based method can be applied to characterize and detect cells of diverse refractive indices caused by any other reason, not limited to apoptosis.

## 4. Conclusions

We proposed and investigated an efficient method for apoptosis monitoring of tumor cells (A549 and HeLa as examples) at microscopic scale based on dynamic laser tweezers. This approach exhibits the capability of detecting antitumor drug which caused slight variation of the cells through the trapping efficiency value, with advantages of more convenient, microscale, label-free, and real time detection than traditional methods. Our works verify that this method can be a potentially important complementary tool in the study of clinical scientific researches for providing the basis of antitumor drug applications. The proposed system might be expanded to any other anticarcinogens, which need further research. Our next objective would be focused on validating the method in a randomised cohort of patients, with a systematic follow-up, to extend and systematise such an assay to other chemotherapeutic agents, which could provide the basis of anticancer drug dosages and save more lives eventually.

## References

[1] Taylor RC, Cullen SP, Martin SJ (2008). Apoptosis: controlled demolition at the cellular level. *Nature Reviews Molecular Cell Biology*.

[2] Saraste A, Pulkki K (2000). Morphologic and biochemical hallmarks of apoptosis. *Cardiovascular Research*.

[3] Lowe SW, Lin AW (2000). Apoptosis in cancer. *Carcinogenesis*.

[4] Kasibhatla S, Tseng B (2003). Why target apoptosis in cancer treatment?. *Molecular Cancer Therapeutics*.

[5] Gerl R, Vaux DL (2005). Apoptosis in the development and treatment of cancer. *Carcinogenesis*.

[6] Wei X, Tan Y, Chen Y Monitoring circulating apoptotic cells by in-vivo flow cytometry.

[7] Ntziachristos V, Schellenberger EA, Ripoll J (2004). Visualization of antitumor treatment by means of fluorescence molecular tomography with an annexin V-Cy5.5 conjugate. *Proceedings of the National Academy of Sciences of the United States of America*.

[8] Green AM, Steinmetz ND (2002). Monitoring apoptosis in real time. *Cancer Journal*.

[9] Shi H, Kwok RT, Liu J, Xing B, Tang BZ, Liu B (2012). Real-time monitoring of cell apoptosis and drug screening using fluorescent light-up probe with aggregation-induced emission characteristics. *Journal of the American Chemical Society*.

[10] Moritz TJ, Taylor DS, Krol DM, Fritch J, Chan JW (2010). Detection of doxorubicin-induced apoptosis of leukemic T-lymphocytes by laser tweezers Raman spectroscopy. *Biomedical Optics Express*.

[11] Mulvey CS, Sherwood CA, Bigio IJ (2009). Wavelength-dependent backscattering measurements for quantitative real-time monitoring of apoptosis in living cells. *Journal of Biomedical Optics*.

[12] Kasili PM, Vo-Dinh T (2005). Optical nanobiosensor for monitoring an apoptotic signaling process in a single living cell following photodynamic therapy. *Journal of Nanoscience and Nanotechnology*.

[13] Ashkin A, Dziedzic JM, Bjorkholm JE, Chu S (1986). Observation of a single-beam gradient force optical trap for dielectric particles. *Optics Letters*.

[14] Guck J, Schinkinger S, Lincoln B (2005). Optical deformability as an inherent cell marker for testing malignant transformation and metastatic competence. *Biophysical Journal*.

[15] Chiou PY, Ohta AT, Wu MC (2005). Massively parallel manipulation of single cells and microparticles using optical images. *Nature*.

[16] Fazal FM, Block SM (2011). Optical tweezers study life under tension. *Nature Photonics*.

[17] Bao G, Suresh S (2003). Cell and molecular mechanics of biological materials. *Nature Materials*.

[18] Guck J, Ananthakrishnan R, Mahmood H, Moon TJ, Cunningham CC, Käs J (2001). The optical stretcher: a novel laser tool to micromanipulate cells. *Biophysical Journal*.

[19] Gu M, Kuriakose S, Gan X (2007). A single beam near-field laser trap for optical stretching, folding and rotation of erythrocytes. *Optics Express*.

[20] Mohanty SK, Uppal A, Gupta PK (2004). Self-rotation of red blood cells in optical tweezers: prospects for high throughput malaria diagnosis.. *Biotechnology Letters*.

[21] Loehrer PJ, Einhorn LH (1984). Cisplatin. *Annals of Internal Medicine*.

[22] Reedijk J, Lohman PHM (1985). Cisplatin: synthesis, antitumour activity and mechanism of action. *Pharmaceutisch Weekblad—Scientific Edition*.

[23] Boulikas T, Vougiouka M (2003). Cisplatin and platinum drugs at the molecular level. *Oncology Reports*.

[24] Ashkin A (1992). Forces of a single-beam gradient laser trap on a dielectric sphere in the ray optics regime. *Biophysical Journal*.

[25] Kundu RK, Cohen IM (2002). *Fluid Mechanics*.

[26] Wu X, Zhang Y, Min C, Zhu S, Feng J, Yuan XC (2013). Dynamic laser tweezers based assay for monitoring early drug resistance. *Laser Physics Letters*.

[27] Yuan X, Zhang Y, Cao R, Zhao X, Bu J, Zhu S (2011). Dynamic steering beams for efficient force measurement in optical manipulation. *Chinese Optics Letters*.

